# The Women’s Tennis Association (WTA) Multidisciplinary Education and Treatment Protocol for the Female Athlete Triad (1996–2022)

**DOI:** 10.3390/sports13070205

**Published:** 2025-06-25

**Authors:** Emily A. Ricker, Kristen J. Koltun, Carol L. Otis, Anna S. Peavler, Mary Jane De Souza

**Affiliations:** 1 Consortium for Health and Military Performance, Department of Military and Emergency Medicine, F. Edward Hébert School of Medicine, Uniformed Services University, 4301 Jones Bridge Rd., Bethesda, MD 20814, USA; emily.ricker.ctr@usuhs.edu; 2 Henry M. Jackson Foundation for the Advancement of Military Medicine, Inc., 6720A Rockledge Dr., Bethesda, MD 20817, USA; 3 Women’s Health and Exercise Lab, Department of Kinesiology, Penn State University, 104 Noll Laboratory, University Park, PA 16802, USA; kjk116@pitt.edu; 4 Neuromuscular Research Laboratory/Warrior Human Performance Research Center, Department of Sports Medicine and Nutrition, University of Pittsburgh, 3860 S. Water St., Pittsburgh, PA 15203, USA; 5 Development Advisory Panel, The Women’s Tennis Association, St. Petersburg, Fl 33701, USA; carolotis921@gmail.com; 6 Performance Health Department, The Women’s Tennis Association, St. Petersburg, FL 37701, USA; apeavler@wtatennis.com

**Keywords:** energy deficiency, low energy availability, nutrition, body image, amenorrhea, bone health, injury prevention, coaching, player monitoring

## Abstract

Elite female tennis players are among those at high risk for developing the Female Athlete Triad (Triad), characterized by three interrelated conditions: energy deficiency/low energy availability, menstrual dysfunction, and low bone mineral density. From 1996 to 2022, the Women’s Tennis Association (WTA) developed and implemented prevention, education, and management plans for female athletes at risk for, or exhibiting symptoms of, the Triad. This article reviews the WTA Triad protocol, developed in 2018 and utilized through 2022, in collaboration with subject matter experts in the Women’s Health and Exercise Laboratory at The Pennsylvania State University. The WTA Triad protocol (1996–2022) includes prevention and management programs implemented by a multidisciplinary Performance Health Team to include screening for “red flags” during annual physicals or upon clinical presentation of a menstrual problem, bone disorder, or nutritional concern; targeted education for players, coaches, and other support team members with handouts and lectures on nutrition and body image to prevent energy deficiency; and a multidisciplinary protocol to guide treatment and return-to-play decisions. Other sport governing bodies can adopt similar multi-layered programs and practices for their athletes, coaches, and support teams to educate, screen, manage, and help to prevent the development of the Triad.

## 1. Introduction

Since 1996, the Women’s Tennis Association (WTA), the governing body of the international professional women’s tennis circuit (i.e., WTA Tour), has been among the first international women’s sports organizations to develop and implement comprehensive prevention, education, and treatment plans for female athletes with components of the Female Athlete Triad (Triad), the syndrome encompassed by the following three interrelated conditions: (1) energy deficiency/low energy availability (ED/LEA) with or without an eating disorder, (2) menstrual dysfunction (e.g., functional hypothalamic amenorrhea), and (3) low bone mineral density (BMD) [1,2]. This article briefly summarizes the basic physiology underlying the Triad and subsequently reviews the WTA Performance Health programs and practices created to educate, prevent, screen, and manage professional female tennis athletes at risk of or exhibiting symptoms of the Triad. These specific practices have been expanded upon since 2022, with emerging research, but for the purpose of this article, the focus will be on the Triad and the WTA practices from 1996 to 2022.

### 1.1. History of the Female Athlete Triad

The Triad was first defined by an expert panel in 1992 [3], with the first official position stand published in 1997 [4] and updated in 2007 [2]. Causal evidence obtained through human [5,6,7] and animal studies [8,9] has demonstrated that ED/LEA is the underlying cause of menstrual disturbances and poor bone health in women with the Triad [1,2]. The Triad affects 16–60% of exercising women [10]; certain subpopulations of athletes, such as those who participate in sports that emphasize leanness for competitive or aesthetic purposes, can be at an elevated risk [11,12,13,14]. In 2014, the International Olympic Committee introduced a new model, Relative Energy Deficiency in Sports (REDs), which proposed an expanded model portraying additional physiological and performance effects of ED/LEA (e.g., immunological, cardiovascular, etc.) [15,16]. The elements of the Triad model (energy status, reproductive function, and bone health) continue to be the REDs outcomes with the highest prevalence and the most evidence supporting a causal relationship between energy and clinical outcomes [17,18,19]. As such, the WTA focused on preventing, screening for, diagnosing, and treating the Triad in its athletes from 1996 to 2022 due to the clinical importance and urgency of early intervention to prevent deleterious outcomes.

### 1.2. The Basic Physiology Underlying the Triad

As mentioned, ED/LEA is the underlying cause of the Triad. ED/LEA occurs when energy intake (i.e., calories from food eaten) is insufficient to meet the demands of energy expenditure, including that of exercise. In response to the energy “mismatch,” metabolic adaptations occur to conserve energy and preserve functions vital for survival (e.g., thermoregulation, locomotion, cellular maintenance) at the expense of less vital functions (e.g., reproduction, growth) [20]. ED/LEA can result from any behaviors that result in inadequate caloric intake: disordered eating, clinical eating disorders, unintentional undereating, or intentional dietary restriction without disordered eating/eating disorders [1]. With respect to reproduction, menstrual dysfunction in younger women can present as primary amenorrhea, or failure to achieve menarche by age 15 [21]. In women who have achieved menarche, menstrual disturbances can range from subclinical, in the case of luteal phase defects or anovulatory cycles [22,23,24], to clinical, in the case of functional hypothalamic amenorrhea (absence of menses for ≥90 days) or oligomenorrhea (long, irregular cycles, 36–89 days). These menstrual disturbances are accompanied by underlying suppression in ovarian steroid hormones, namely estrogen and progesterone, with the severity of menstrual disturbance proportional to both the degree of hormone suppression and degree of energy deficiency [25]. ED/LEA impacts bone health through direct and indirect mechanisms. Directly, insufficient energy results in uncoupling of bone turnover such that bone resorption outpaces bone formation, which may ultimately lead to bone loss [26,27,28,29]. Indirectly, hypoestrogenism in response to ED/LEA impacts bone, as estrogen plays a role in preventing excess bone resorption and promoting bone formation [30]. Therefore, the combination of both ED/LEA and menstrual disturbances can be especially harmful to bone [31,32]. Importantly, the Triad is considered a spectrum disorder, as each component (energy status, reproductive function, and bone health) can range from optimal to subclinical to pathological clinical endpoints, and the presence of any one clinical (e.g., osteoporosis) or subclinical (e.g., luteal phase defect) condition may warrant further investigation and an eventual diagnosis [1,2].

### 1.3. The Triad in Tennis Athletes

Elite female tennis players are exposed to media pressures (portraying a thin, idealized body image) and pressure from coaches or other stakeholders to lose weight (in a mistaken belief that “thinner is faster”). These athletes are among those at high risk for the development of disordered eating, ED/LEA, and the Triad [33,34,35]. Although there are only a few studies assessing Triad components in elite tennis players, one study described a high prevalence of Triad-related conditions in 24 adolescent Brazilian tennis players: 91.7% of the athletes met criteria for disordered eating and/or ED/LEA, 33.3% for menstrual irregularities, and 25% for low bone mass [36]. Further, a greater percentage of tennis players than sedentary controls presented with Triad components, and one athlete presented with all three components of the Triad [36]. Similarly, in a study of 26 elite French professional tennis players, 46% had disordered eating attitudes based on standardized questionnaire assessments [37]. Additionally, those with disordered eating attitudes had lower energy intake and body mass index (BMI) and higher state anxiety than in the group without disordered eating attitudes [37]. In addition to health concerns relating to menstrual dysfunction (hypoestrogenism) and suboptimal BMD, ED/LEA and the Triad are also associated with increased risk of injury, such as stress fracture [38], and hindered sport performance [39].

### 1.4. Development of a WTA Triad Protocol

Identifying athletes at risk for, or exhibiting symptoms of, the Triad was crucial in order to intervene early and properly treat, manage, and prevent the progression of the condition, ultimately preserving optimal health and performance in the athlete. From 1996 to 2022, the WTA took a proactive approach to Triad prevention and management. In 1996, an independent scientific commission found that young female players needed support from minimum age rules for professional sport participation and education programs to help ease athletes into the international arena of professional women’s tennis [40]. The approach has evolved to include protocols and programs managed by the WTA Performance Health Team (PHT). Education specific to the Triad is implemented by a PHT consisting of primary health care providers (PHCPs, including athletic trainers and physical therapists), massage therapists, mental health care providers, medical advisors, and sport dietitians. Stakeholders (players, coaches, parents, and agents) in the WTA tennis community digitally receive various performance health education topics. Additionally, players and their coaches who are entering the WTA are required to complete education modules on nutrition, hydration, injury prevention, and other health-related topics. Individual education and intervention for the Triad, specifically, began with the WTA player being screened for Triad “red flags” [1,2] (Table 1) at her annual physical and also upon clinical presentation of a menstrual problem, bone disorder, or nutritional concern. These red flags include a combination of research-documented symptoms of the Triad and indicators deemed relevant by those with clinical experience treating athletes with the Triad. This article summarizes the Triad programs and policies in place in the WTA from 1996 to 2022 and provides a successful example for other sporting organizations developing protocols to prevent and manage the Triad and other related syndromes.

## 2. Triad Prevention and Education

**Figure 1:**
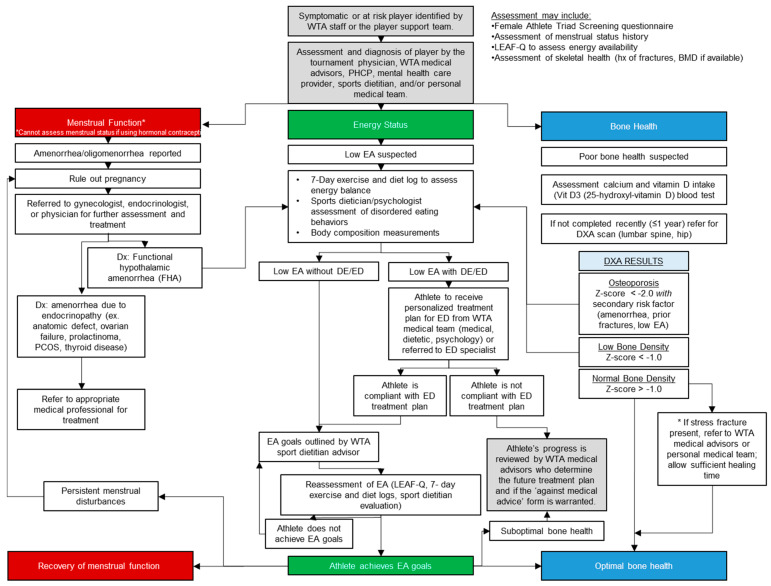
WTA clinical decision-making tree. This diagram is part of the WTA protocol and is used by the WTA multidisciplinary team when there is suspicion of red flags or risk factors for the Female Athlete Triad. Energy availability (EA); eating disorder (ED); disordered eating (DE) Reprinted with permission from the Women’s Tennis Association.

The WTA approach to Triad prevention involves first educating and empowering the PHT with specific protocols and policies written in consultation with experts in the field and approved by WTA legal. In 2018, a Triad protocol was developed for the WTA in collaboration with subject matter experts in the Women’s Health and Exercise Laboratory at The Pennsylvania State University. The protocol is summarized in a clinical decision-making tree (Figure 1) that outlines procedures for assessment and diagnosis of the three interrelated conditions of the Triad, i.e., menstrual function, energy status, and bone health. All WTA PHCPs are expected to know, understand, and implement the protocol. The protocol may also be shared with a player’s individual physician and WTA tournament physicians around the world to further educate physicians on the WTA Triad treatment process.

Additionally, members of the PHT are trained in part by the WTA University, an online private learning platform, covering topics such as nutrition, female health, and bone stress injuries to ensure they are equipped with the necessary knowledge and tools to assess athletes presenting with aspects of the Triad. Players and support team members receive targeted education with handouts and lectures on health, nutrition, and body image. In addition, an online series, titled “*Physically Speaking*”, was also developed by the PHT and leading medical providers to provide more in-depth information on health topics and can be given to athletes on an as-needed basis. Another WTA educational strategy includes informational signs posted at every tournament on topics such as hydration techniques, proper recovery routines, and fueling for matches. Posting educational and referral information in highly viewed areas, such as on the back of bathroom doors in tournament locker rooms, is another method used and mentioned by players as a useful avenue for education.

Since ED/LEA is the causative factor underlying the Triad, it is essential that education and prevention strategies focus on ensuring the athlete and her support team are aware of the importance of performance fueling. Signs specific to nutrition and fueling are purposefully posted in the dining areas and often spark the interest of players, coaches, and fitness trainers. Athletes and player support team members can also receive specific dietary advice from the on-site WTA sports dietitians and have individualized meal plans created to help meet energy requirements. Notably, sports dietitians play an integral role in Triad prevention by enabling players to adequately fuel to meet their energy expenditure needs. For tournaments, 7-day meal plans are developed that include low-fat and high-carbohydrate choices, lean proteins, vegetarian and gluten-free options, healthy snacks, chocolate milk or recovery drinks, and international seasonings and condiments to ensure that athletes are fueling adequately and to their taste. There may be added fueling challenges for elite athletes who travel internationally, for which strategies have been developed by WTA sports dietitians. For example, if an athlete is traveling to a foreign country that may not have local cuisine tailored to an athlete’s diet or if an athlete is worried about travel and food selections, WTA sports dietitians can provide one-on-one consultations to give tips on travel snacks and finding healthy food choices in different countries and provide a balanced meal plan for the athlete.

Body image concerns may also lead to suboptimal nutrition in elite athletes, especially due to perceived media pressure [45]. To combat this problem, the WTA provides media training to inform players about how social and mass media messages are constructed. For example, media may portray a thin ideal body image that can lead athletes to develop a high drive for thinness and intentionally restrict energy intake. Media training can empower the athlete with the knowledge needed and provide the skills to respond to inappropriate or unhealthy messages around body image [46].

## 3. Screening and Diagnosis of the Triad in Tennis Athletes

### 3.1. Screening

Screening and early detection among athletes at risk for the development of any component of the Triad is critical and requires a multidisciplinary health care team approach [47,48], which can include tournament physicians, medical advisors, PHCPs, mental health and performance team members, sports dietitians, and/or an athlete’s personal medical team. In 1996, the WTA instituted annual mandatory physicals completed on-site by PHCPs, medical advisors, and sports dietitians that include Triad screening questions to detect “red flags” (Table 1). Specific questions currently used to screen for the Triad include questions related to menstrual regularity, hormonal medication use, weight concerns, eating disorder history, and stress fracture history. As of 2022, annual physicals also include dual energy X-ray absorptiometry (DXA) scanning to assess BMD and body composition [49]. Triad screening can also occur when an athlete presents with any of the Triad symptoms, such as a stress fracture or irregular menstrual cycle, or is identified by WTA staff or the player support team as being at risk for the Triad. For example, when an athlete presents in the training room with a possible bone stress injury, the PHT uses the WTA Triad protocol, specifically the decision tree (Figure 1), to confirm that all necessary steps are followed, including appropriate initial questioning, determining possible need for referral to a specialist, and athlete education. Through three intersecting diagrams, decision-making and treatment strategies should be made in a comprehensive manner to address all components of the Triad.

### 3.2. Diagnosis

Diagnosing each component of the Triad can be achieved by following recommendations published by the Female Athlete Triad Coalition [1]. Below, the recommendations are summarized, followed by a detailed description of the specific procedures implemented by the WTA for their athletes.

#### 3.2.1. Energy Deficiency/Low Energy Availability

There are several methods for assessing ED/LEA. Initial indications of LEA include recent weight loss and a BMI < 17.5 kg/m^2^ or <85% of expected body weight in adolescents (approximately age 10–19 years). However, weight stability or estimates of energy balance alone should not be used to diagnose ED/LEA, specifically, since physiological functions can be suppressed in the face of LEA and weight may remain stable, as has been observed in amenorrheic athletes [50]. Other measures can include those used specifically to assess physiological signs of a chronic energy deficiency and include low total triiodothyronine (TT_3_) hormone levels [51,52], reduced resting metabolic rate (RMR) [53], and/or a ratio of measured to predicted resting metabolic rate (mRMR/pRMR) of less than a given threshold, which varies by RMR prediction method (i.e., Harris–Benedict ratio and Cunningham_1980_ ratio < 0.90, Cunningham_1991_ ratio < 0.92, DXA ratio < 0.94) [51].

EA, specifically, can be calculated based on food intake and energy expenditure records according to the following equation:

$$EA=\left[energy\;intake\;\left(kcal\right)-exercise\;energy\;expenditure\;\left(kcal\right)\right]/Fat\;Free\;Mass\;(FFM)\;(kg).$$

Physically active women should aim for at least 45 kcal/kg FFM/day to ensure adequate EA for all physiological functions [1]. A 3- to 7-day diet and exercise log can be used to assess energy intake and exercise energy expenditure; however, accurate dietary intake assessments can be difficult to acquire due to issues such as under- or over-reporting of intake, modified intake during reporting periods, and imprecise reporting of portion sizes [54,55]. To determine exercise energy expenditure, athletes may use heart rate monitoring or estimate exercise energy expenditure based on the Compendium of Physical Activities, where [56,57]

$$Exercise\;Energy\;Expenditure\;\left(kcal\right)=METs\left(kcal*{kg}^{-1}*{hr}^{-1}\right)\;x\;weight\left(kg\right)\;x\;duration\left(hrs\right).$$

To assess FFM, a total body DXA scan is optimal; however, other field measures can be substituted where necessary with varying degrees of error (air plethysmography, hydrostatic weighing, bioelectrical impedance, skinfold measurements) [58]. Importantly, in cases where an athlete may be at risk for nutrient deficiencies, a registered sports nutritionist should complete a comprehensive nutrition assessment.

The aforementioned methods of assessing EA may be difficult to obtain from a traveling tennis player; therefore, when ED/LEA or the Triad is suspected, WTA athletes meet in person or virtually with a licensed sports dietitian to evaluate their food intake, training routine, and recovery techniques. The athlete is asked to fill out an initial dietary consult and have the sports dietitian review the form. Upon completion of the initial dietary consult, the athlete may be asked to fill out a food diary and exercise log to monitor food intake and exercise energy expenditure during 7 days of practice and competition. An initial dietary consult combined with a 7-day food diary provides greater insight into eating behaviors and practices compared to either component alone, as estimating energy expenditure needs is essential to understanding and calculating EA and dietary intake needs. This consult is also a time when recovery snacks/drinks can be suggested. Additional components of Triad diagnosis may include body composition measurements and/or assessment of disordered eating behaviors by a sports dietitian or mental health practitioner. If appropriate, the athlete is also educated on the consequences of ED/LEA, such as fatigue, poor recovery, poor healing, bone loss, and impaired performance.

#### 3.2.2. Menstrual Disturbances

Athletes who present with amenorrhea or oligomenorrhea are assessed by the PHCPs and then referred for further evaluations. The athlete is first evaluated to rule out pregnancy and other endocrinopathies, such as thyroid dysfunction, hyperprolactinemia or prolactinoma, primary ovarian insufficiency, hypothalamic and pituitary disorders, and hyperandrogenic disorders, such as polycystic ovary syndrome (PCOS) [43]. Amenorrhea secondary to ED/LEA is a diagnosis of exclusion, and the most common alternative causes can often be identified after a thorough medical history, physical examination, pregnancy test, and evaluation of labs such as thyroid-stimulating hormone, follicle-stimulating hormone, and prolactin [43]. In cases where there is evidence of androgen excess (i.e., hirsutism, acne, or androgenic alopecia), laboratory tests of total and free testosterone and dehydroepiandrosterone and its sulfate (DHEA-S) may be warranted, as well as a pelvic ultrasound to confirm polycystic ovaries or rule out virilizing ovarian tumors [59]. A position stand detailing functional hypothalamic amenorrhea diagnosis was published by the Endocrine Society in 2017 [43].

If an athlete is using hormonal contraception, it is not possible to assess menstrual status, as combined hormonal contraceptives suppress menstrual function. Many hormonal contraceptive options, including oral contraceptives (“the pill”), the transdermal patch, and the vaginal ring, are cyclically administered such that every month, the athlete will experience withdrawal bleeding during a hormone-free (placebo) week. Other types of hormonal contraception, such as progesterone intrauterine devices (IUDs) and/or progesterone subdermal (Nexplanon), can suppress menses altogether. As such, bleeding patterns that present in women using hormonal contraception are not reflective of endogenous reproductive hormone profiles and, therefore, are not indicative of an athlete’s menstrual status or energy status [1,60]. In fact, withdrawal bleeding during hormone-free (placebo) phases of contraception or menstrual suppression due to continuous use of combined hormonal contraception pills may actually mask menstrual disturbances that are an important sign of ED/LEA and menstrual status. If an athlete who is using hormonal contraception is suspected to have ED/LEA, clinicians should take the appropriate steps to screen the athlete’s nutritional status. Likewise, because hormonal contraception can mask ED/LEA-induced menstrual disturbances, hormonal contraception is not advised as a first-line strategy to treat menstrual disturbances in athletes. Hormonal contraceptives deliver suboptimal concentrations of estrogen, and the estrogen delivered (ethinyl estradiol) is not identical to the estrogen produced naturally (17-β estradiol). Further, oral administration of hormonal contraceptives can suppress the production of hormones important for bone, including insulin-like growth factor-1 (IGF-1), in a process called hepatic first-pass effects [61]. Together, the low-dose non-physiologic form of estrogen and first-pass effect of oral contraceptives may explain the lack of efficacy to improve BMD in athletes with pre-existing menstrual disturbances [62,63]. If athletes are in need of contraception, the risks and benefits of all available options should be weighed so that informed choices can be made for the athlete’s health. The Female Athlete Triad Coalition has provided recommendations about very specific circumstances when to use estrogen therapy, which include worsening BMD or ongoing stress fractures, and involves transdermal administration of 17-beta estradiol with oral cyclic progesterone [1].

#### 3.2.3. Low Bone Mineral Density

DXA scans are primarily used to evaluate BMD and can also be used to measure total body composition (fat mass, lean body mass, FFM). For adult athletes ≥ 20 years old, BMD should be assessed at weight-bearing sites (lumbar spine, total hip, and femoral neck). For adolescent athletes and women < 20 years old, bone mineral content (BMC) and BMD should be assessed at the lumbar spine and, if possible, the whole body less the head (otherwise, the whole body) [1]. DXA scans provide results in two scores: Z-score and T-score. The Z-score is the number of standard deviations a patient’s BMD differs from the average BMD of their age, sex, and ethnicity. The T-score is a comparison of a patient’s BMD to that of a healthy thirty-year-old of the same sex and ethnicity and is used in postmenopausal women because it better predicts risk of future fracture. Premenopausal women should have the Z-score assessed for the appropriate age, sex, and ethnicity [64]. The Female Athlete Triad Coalition recommends that athletes should receive DXA scans if they present with any of the following: (1) at least one “high-risk” Triad risk factor, (2) at least two “moderate-risk” Triad risk factors, (3) a history of at least one nonperipheral or at least two peripheral long bone traumatic (non-stress) fractures in the face of one or more moderate- or high-risk Triad risk factors, and/or (4) at least 6 months of use of medication that may impact bone [1]. “High-risk” Triad risk factors include a history of DSM-5-diagnosed eating disorder; BMI ≤ 17.5 kg/m^2^, <85% estimated weight, or recent weight loss of ≥10% in 1 month; menarche ≥ 16 years old; current or history of <six menses in 12 months; two prior stress fractures, one high-risk stress fracture, or a low-energy nontraumatic fracture; and prior Z-score < −2.0 after at least 1 year from baseline DXA. “Moderate-risk” Triad risk factors include a current or history of disordered eating for ≥6 months; BMI between 17.5 and 18.5 kg/m^2^, <90% estimated weight, or recent weight loss of 5–10% in 1 month; menarche between 15 and 16 years old; current or history of 6–8 menses in 12 months; one prior stress reaction/fracture; and prior Z-score between −1.0 and −2.0 (after at least a 1-year interval from baseline DXA) [1]. The recommendations for diagnosing low BMD and osteoporosis follow the guidelines from the International Society for Clinical Densitometry (ISCD), such that a combination of low BMD and a clinically significant fracture history are required for an osteoporosis diagnosis in children and adolescents, and premenopausal women require both low BMD and a secondary cause of osteoporosis, with the caveat that low BMD in adolescent and young adult female athletes in weight-bearing sports (such as tennis) can be defined as a Z-score < −1.0, rather than the typical threshold of Z-score < −2.0 [1,42,65].

When a WTA athlete is identified as having low BMD or at risk of having low BMD, her case is discussed with the WTA medical advisors to determine the appropriate next steps, including further diagnostic testing. Assessments of calcium and vitamin D are conducted via a food diary and blood test, respectively, and, if not completed within the past year, the athlete is referred for DXA screening. Additionally, if a bone stress injury is present, the athlete is referred to WTA medical advisors or her personal medical team for evaluation of underlying causes and treatment. She is also referred for a dietary consult, which includes food diary and exercise log completion. These data are reviewed by the sports dietitian and shared with the athlete’s medical team in order to comprehensively assess if the athlete is receiving the adequate energy and nutrients to support bone health.

Beginning at the 2022 BNP Paribas Open in Indian Wells, CA, WTA athletes are offered BMD screening of the lumbar spine, total hip, and forearms (Hologic Horizon^®^, Hologic Inc., Bedford, MA, USA) during their annual physical examination [49]. DXA screening was used to establish baseline BMD and body composition values for WTA athletes, which, together with clinical history, may be useful in the evaluation and treatment of the Triad. Long term, the WTA intends to use the data collected from the baseline DXA scans to establish normal reference ranges for WTA professional tennis athletes and potentially provide predictive models for injury risk. More research will be needed to see if these normative values can be generalized to other weight-bearing sports.

The WTA Triad protocol (1996–2022) of assessing BMD Z-scores from DXA follows recommendations from the Female Athlete Triad Coalition and the ISCD and aligns with research demonstrating low BMD as a risk factor for bone stress injuries in female athletes (e.g., [38]). An additional measure of bone health that can now be derived from DXA imaging is Trabecular Bone Score (TBS), which evaluates grayscale variation in lumbar spine DXA images as a surrogate for bone microarchitecture [66]. This capability was developed in an effort to improve fracture risk prediction from DXA and has been used effectively in older postmenopausal women, in whom lower TBS scores are associated with increased risk of osteoporotic fracture [66]. However, in younger, premenopausal women, TBS has not demonstrated consistent associations with fracture risk, including osteoporotic fracture, all fractures, and stress fracture/bone stress injury [66,67,68]. For instance, in a study of male and female adults ages 20–39 years, TBS score was not associated with incident fracture risk [67]. However, in a sample of collegiate female athletes, a lower TBS score was associated with increased odds of bone stress injury, especially for trabecular-rich bone injury sites, but including TBS in the model did not improve the prediction of injury risk compared with the model that included spinal BMD alone [68]. Until more evidence demonstrates a consistent and reliable utility of TBS to improve bone health evaluation and risk assessment in younger athletic populations, the WTA supports and recommends other sporting organizations continue to abide by ISCD recommendations to only consider TBS in adults ages 40 years and older [66].

## 4. Treatment and Management

**Figure 2:**
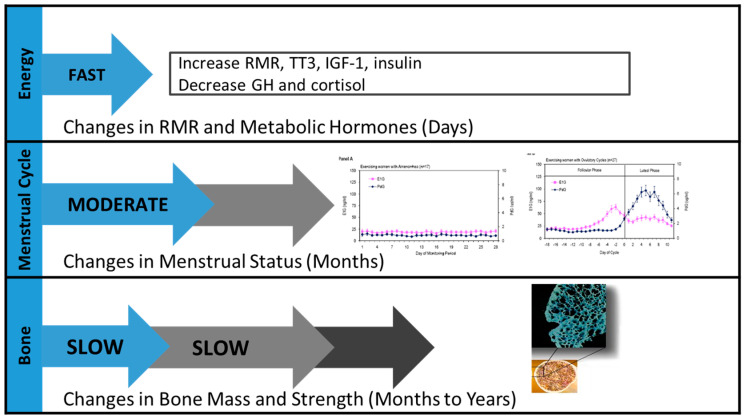
Recovery from the Female Athlete Triad. The three components of the Triad recover at different rates with the appropriate treatment.

Recovery from each component of the Triad may occur at differing rates when an appropriate treatment strategy of increasing energy intake is initiated (Figure 2) [1]. Recovery of energy status is typically observed after days or weeks of increased energy intake and/or decreased energy expenditure, but the rate of recovery is dependent upon the degree of ED/LEA and the presence of disordered eating or eating disorder behaviors that require intervention. Recovery of menstrual status may be observed after months of increased energy intake and/or decreased energy expenditure, which improves energy status [69,70]. Recovery of BMD may not be observed until one or more years after recovery of energy status and until menstrual recovery has been achieved for several years [1,71]. It is also possible that bone may not fully recover if energy deficiency and menstrual dysfunction are prolonged, especially during the time of peak bone mineral accrual (adolescence to young adulthood) [72,73]. In a recently published randomized controlled trial called REFUEL, increasing energy intake by approximately 350 kcal/day for 12 months successfully resumed menstrual frequency in amenorrheic and oligomenorrheic athletes but was not adequate to significantly improve estrogen exposure or BMD, despite an increase in body weight, fat mass, and TT_3_ [69,72]. More data are required at higher energy intakes (>350 kcal/d) to determine the adequacy of increased refueling in athletes with severe menstrual disturbances on recovery of BMD.

The best practice for management of the Triad is demonstrated by the incorporation of a multidisciplinary team. Player support team members who may become involved, at the discretion of the overseeing physician and athlete, may include the athlete’s coach, family members, and other professionals involved in the athlete’s career [1]. To approach a traveling professional tennis athlete about suspected Triad conditions, especially one linked to an eating disorder, body image, or menstrual cycle disturbance, it is necessary to have her confidence and trust and ensure confidentiality. To be a trusted team member, the WTA multidisciplinary team must not only be knowledgeable in their field, but they must also understand and be part of the world of the professional tennis player. The WTA therefore schedules members of the PHT to be present in the same environment as the player. The PHCPs, massage therapists, and mental health care providers travel with the players, living the same life of travel and unpredictable schedules. These professionals have a constant presence in each tournament training room, gaining trust from players while providing treatment and player education on an as-needed basis and as part of the athlete’s daily experience. An athlete is assigned to one PHCP for continuity when the player is not traveling.

If a WTA athlete has ED/LEA or any component of the Triad, early recognition and action are taken. The first approach is for the PHCP to meet with the athlete to reinforce the following:

- Regular periods can be an indicator (“barometer”) of good health and adequate nutrition.
- Estrogen and progesterone are important hormones that regulate the female reproductive system. These hormones also help build and maintain strong bones.
- Menstrual disorders may contribute to difficulty conceiving or maintaining a pregnancy, but you could still get pregnant even if you are not getting a period. If that athlete is sexually active, contraception is advised.
- Not having a period could mean the athlete has an underlying health condition that needs to be investigated.
- Consequences of long-term amenorrhea, like low BMD, may not be completely reversible; thus, early diagnosis and intervention are critical.

To facilitate athlete participation in the treatment plan, the multidisciplinary team must develop a therapeutic alliance with the athlete [1]. The development of any Triad treatment plan should include and consider the goals of the athlete, her unique diet and training regimen, any coexisting conditions, mental health issues, and a systemic approach for monitoring changes. The overseeing physician must also take into account all aspects of the athlete’s health when making the decision regarding safe return to play following diagnosis of any aspect of the Triad. Returning to competition too soon can be detrimental to an athlete’s season and career longevity. Thus, return-to-play criteria include functional testing, approval from the overseeing dietitian and sports psychologist as necessary, and acknowledgement from the athlete and her team that maintaining communication with the WTA PHT throughout her career is critical to her overall well-being and success. Professionals are encouraged to reference the 2014 Female Athlete Triad Coalition Statement on Treatment and Return to Play for detailed guidelines to this effect [1].

## 5. Future Directions and Protocol Modifications

Since the development and use of the WTA Triad protocol from 1996 to 2022, Triad science and associated clinical practice have continued to evolve. The following recommendations are provided as suggested modifications to the Triad protocol for other sporting organizations that wish to follow the example set forth by the WTA in creating a Triad protocol for their athletes.

Since weight/BMI can remain stable even in the presence of energy deficiency, we emphasize the use of serial measures of other markers of metabolic compensation, such as changes in metabolic hormones like TT3 or decreasing weight/BMI or body fat percentage. These objective metabolic biomarkers may be more reliable than calculating EA based on food and exercise logs, especially considering how food logs are prone to reporting inaccuracies [74] and accurate calculation of exercise energy expenditure requires sensitive, often expensive technology [75]. Regarding assessment of menstrual function and management of menstrual disturbances, more recent research has demonstrated that although previously amenorrheic women may resume menses with dietary intervention, menstrual quality may still be hampered, with many “recovered” women demonstrating anovulatory cycles and suppressed ovarian hormone profiles [69,76]. Thus, it is suggested that women continue to be monitored to ensure multiple consecutive cycles of normal length are achieved, as this more robust level of recovery is associated with improved cycle quality [76]. The WTA’s continued efforts to assess BMD in their athletes is significant, as low Z-scores are a risk stratifier, and increased risk of bone stress injury is an important clinical outcome associated with the Triad [38,77,78,79]. This is particularly true for higher-grade bone stress injury and injury to trabecular bone sites, as return to play is often longer for these injuries [80]. As bone imaging technologies evolve to allow for more high-resolution, sensitive assessments of bone structure and microarchitecture, we recommend sporting organizations adopt such advances as they become clinically available and recommended [81,82]. Further, the WTA’s use of multidisciplinary teams to prevent and manage the Triad in tennis athletes, especially the use of mental health professionals as needed, is a critical layer of this successful WTA program.

## 6. Conclusions

The WTA has been among the first international women’s sports to develop and implement comprehensive education, recognition, and treatment plans for female athletes with components of the Triad in the 1996–2022 protocols. The programs are athlete-centered and coordinated by the PHCPs, who travel with the players. Comprehensive annual physicals screen for “red flags” for components of the Triad, and if one is recognized, the athlete is referred for appropriate care from the WTA PHT, which includes physical therapists, athletic trainers, mental health care providers, massage therapists, and sports dietitians and is coordinated with WTA medical advisors and tournament physicians. The PHT providers follow a Triad protocol developed specifically for the WTA athletes, their coaches, and their other support team members, who also receive targeted education on the topics related to the Triad, such as nutrition, menstrual health, body image, and the media.

This comprehensive overview of the 1996–2022 WTA Triad protocol exemplifies successful implementation of the Female Athlete Triad Coalition consensus guidelines [1,2], offering a model for other sporting organizations to optimize care for athletes with the Triad. The 1996–2022 WTA Triad protocol provides specific guidance for caring for elite tennis athletes, tailoring the broad recommendations from numerous professional sporting and medical organizations. For instance, a consensus statement from a collaboration of six major professional associations has published “*Female Athlete Issues for the Team Physician*,” which overviews what team physicians ought to know with respect to female-specific musculoskeletal and medical conditions, including those included in the Triad (low energy availability, disordered eating, eating disorders, menstrual dysfunction, bone health, and bone stress injury) [83]. A similar collaborative consensus statement charges team physicians with the duty of establishing return-to-play processes that do not put the individual at undue risk for injury or illness [84]. The 1996–2022 WTA Triad protocol provides detailed guidance for the care and return-to-play decision-making process for tennis athletes in alignment with these consensus statements. The availability of nutritional professionals and guidance in the 1996–2022 WTA Triad protocol is supported by the National Athletic Trainers’ Association’s published “*Appropriate Medical Care Standards for Organizations Sponsoring Athletic Activity for the Secondary School Age Athlete*,” which includes a standard that “education and counseling (be) provided for athletes on nutrition, hydration, and dietary supplementation” [85]. Similarly, nutrition assistance for the traveling tennis athlete follows the Health Care Guidelines for International Federation Events set forth by the Association of Summer Olympic International Federations (e.g., “…medical teams should ensure adequate and appropriate nutrition at international sporting events. Nutritional requirements should include…provision of a variety of food options to aid athlete recovery…food/menu options that meet the health and specific sports nutrition considerations of the athlete population…sufficient accessibility to support the overall nutrition needs of the athletic group and the logistics around training and competition schedules…” [86].

The WTA is committed to incorporating new and emerging research and science into their practices in order to ensure their players are provided with the highest quality and relevant care. In order to best support their athletes, the WTA PHT has updated its protocol as science and clinical recommendations have evolved. In the future, the WTA plans on providing outcomes based upon the implementation of their new WTA female athlete protocol in hopes of further setting the standard for female athlete care.

## Figures and Tables

**Table 1:** Indicators or “red flags” for each component of the Triad used by the WTA.

Low Energy Availability	Menstrual Disturbances	Low Bone Mineral Density
Fatigue	Long and irregular menstrual cycles of 36–90 days, defined as oligomenorrhea	Family history of osteoporosis
Irritability and mood changes	Complete absence of menstrual cycles, defined as amenorrhea	Stress fracture and/or other secondary clinical risk factors for fracture, previous fractures [41]
Difficulty handling travel or time zone changes	Difficulty conceiving or maintaining pregnancy	Nutrition: low calcium intake and/or low vitamin D status, restrictive calories or recent weight loss, restrictive diets (veganism, vegetarianism, etc.), history of eating disorders
Poor recovery after training	Initiation of hormonal contraceptive use in response to irregular/absent menstrual cycles	Medication: corticosteroid medications for 3+ months [42]
Difficulty healing from minor injuries	Primary amenorrhea (no menses by age 15 years) [21,43,44]	Hormonal deficiencies: amenorrhea (primary and secondary), low testosterone, hypoestrogenism (estrogen is important in building and maintaining bone mass)
History of recent dieting or comments about needing to lose weight		Medical conditions that can reduce bone mass: lupus, cirrhosis, alcoholism, atherosclerosis, thyroid disease, hyperparathyroidism, celiac disease
Restrictive eating patterns, food faddism		
Preoccupation with weight		
Unable to get into “high gear” during training or a match		
Lack of energy or fading in long matches		
Failing to improve performance despite ongoing training		
Changes in menstrual cycle		
Frequent illness		

WTA: Women’s Tennis Association; Triad: Female Athlete Triad.

## Disclaimer

The opinions and assertions expressed herein are those of the author(s) and do not reflect the official policy or position of the Uniformed Services University or the Department of Defense. The contents of this publication are the sole responsibility of the author(s) and do not necessarily reflect the views, opinions, or policies of The Henry M. Jackson Foundation for the Advancement of Military Medicine, Inc. Mention of trade names, commercial products, or organizations does not imply endorsement by the U.S. Government.
